# The Effect of Chloride Anions on Charge Transfer in Dye-Sensitized Photoanodes for Water Splitting

**DOI:** 10.3390/biomimetics4010005

**Published:** 2019-01-16

**Authors:** Iwona Grądzka, Mateusz Gierszewski, Marcin Ziółek

**Affiliations:** Quantum Electronics Laboratory, Faculty of Physics, Adam Mickiewicz University, ul. Umultowska 85, PL-61614 Poznań, Poland; iwona.gradzka@amu.edu.pl (I.G.); mgiersz@amu.edu.pl (M.G.)

**Keywords:** dye-sensitized photoelectrochemical cell, charge transfer, chloride anions, titanium dioxide, ruthenium catalyst, electron transfer, hole-hopping

## Abstract

The photoelectrochemical behavior of dye-sensitized photoelectrochemical cells based on a TiO_2_ layer sensitized with ruthenium components, including an absorber, ruthenium(II)bis(2,2′-bipyridine)([2,2′-bipyridine]-4,4′-diylbis(phosphonic acid)) dibromide (RuP), and a catalyst, ruthenium(II) tris(4-methylpyridine)(4-(4-(2,6-bis((l1-oxidanyl)carbonyl)pyridin-4-yl)phenyl) pyridine-2,6-dicarboxylic acid) (RuOEC), was investigated in the following water-based electrolyte configurations: KCl (pH ≈ 5), HCl (pH ≈ 3), ethylphoshonic acid (pH ≈ 3) with a different KCl concentration, and a standard phosphate buffer (pH ≈ 7). The rate of charge transfer on the photoanode’s surface was found to increase in line with the increase in the concentration of chloride anions (Cl^−^) in the low pH electrolyte. This effect is discussed in the context of pH influence, ionic strength, and specific interaction, studied by cyclic voltammetry (CV) in dark conditions and upon illumination of the photoanodes. The correlations between photocurrent decay traces and CV studies were also observed.

## 1. Introduction

In nature, solar energy is stored in the form of chemical bonds through a natural photosynthesis system. The key elements of natural photosynthesis are light-harvesting by chlorophylls, spatial charge separation after photoexcitation, and catalytic water oxidation or reduction reactions, which lead to the production of nicotinamide adenine dinucleotide phosphate (NADPH)’s redox active hydrogen. Artificial photosynthesis is a bioinspired chemical approach, aiming to emulate the general principles of the natural processes using much simpler man-made materials, and leading to the conversion of solar energy into chemical energy in the form of hydrogen [1].

One of the most widely explored areas of artificial photosynthesis is photoelectrochemical water splitting [2,3]. In the recent trend of water splitting studies, considerable attention has been paid to dye-sensitized photoelectrochemical cells (DSPCs) incorporating ruthenium sensitizers as absorbers and/or catalysts [1,4]. These compounds, as the chromophores, exhibit many attractive features such as strong light absorption in the visible range, appropriate highest occupied molecular orbital (HOMO) and lowest unoccupied molecular orbital (LUMO) energy levels, and relatively long-lived excited state lifetimes, making the efficient charge transfer process possible. Acting as catalysts, ruthenium compounds of specially designed chemical structures are also very promising for obtaining high turnover numbers and high turnover frequencies, which are quantitative criteria for water oxidation catalysts [5,6,7]. On the other hand, new approaches for maintaining long-term stability of DSPCs have been recently explored, since the dyes can be detached from the TiO_2_ surface in a water environment by hydrolysis. The dyes with phosphonic acid anchoring units are considered to be more strongly bonded to the titanium surface than the carboxylic ones [8]. However, they are unstable near and above pH 7 [4]. On the other hand, low water oxidation rate is observed in acidic solutions. Adding a buffer base like PO_4_^3−^ to the electrolyte significantly enhances this process [9], but there is a risk of anation of the ruthenium compounds, increasing with growing buffer concentration, which causes the loss of catalytic effect [10].

Another approach to increase the rate of water oxidation reaction is the addition of Cl^−^ (e.g., from NaCl). The Cl^−^ anion can be oxidized to HOCl by a ruthenium catalyst, then the latter can be oxidized to O_2_. However, this mechanism can work above pH ≈ 5, when Cl_2_ becomes unstable and can disproportionate to anions which act as intermediates for catalytic water splitting [9]. Moreover, because Cl^−^ feature the ability of scavenging holes, they may promote separation of the electron-hole pairs in photoexcited titanium dioxide [11]. On the other hand, Cl^−^ anions can also coordinate toward transition metals, and they may therefore interrupt the catalytic work of the ruthenium catalyst and change the spectral characteristics of the ruthenium dye [12]. Here, we investigate the photocatalytic performance of dye-sensitized photoelectrochemical cells with a ruthenium dye, ruthenium(II) bis(2,2′-bipyridine)([2,2′-bipyridine]-4,4′-diylbis(phosphonic acid)) dibromide (RuP) (Supplementary Figure S1A), acting as a chromophore; and a ruthenium water oxidation catalyst, ruthenium(II) tris(4-methylpyridine) (4-(4-(2,6-bis ((l1-oxidanyl) carbonyl) pyridin-4-yl) phenyl) pyridine-2,6-dicarboxylic acid) (RuOEC) (Supplementary Figure S1B) in a low pH electrolyte conditions with Cl^−^ anions, in order to determine the specific interactions between incorporated species that would affect the charge transfer mechanism.

Photoanodes incorporating ruthenium compounds, including RuP and RuOEC, have been recently investigated for photoelectrochemical water splitting [13,14,15,16,17]. The high catalytic activity of a system, similar to that reported here, working in a typical three-electrode configuration has been evidenced by obtaining high average photocurrent density of c.a. 100 μA cm^−2^ upon illumination of the photoanode. Photoelectrochemical measurements were conducted in a degassed phosphate buffer at pH 7 [13].

In our study, we took into account the charge transfer mechanism occurring on the TiO_2_ surface sensitized with the RuP absorber and the RuOEC catalyst in response to the illumination and voltage biasing. These studies were carried out in water-based electrolytes, including HCl, KCl, ethylphosphonic acid (EPA) solutions, and a standard phosphate buffer, to elucidate the effect of electrolyte ions on the photoelectrochemical response of the titanium dioxide sensitized with ruthenium compounds.

## 2. Materials and Methods

### 2.1. Photoanode Preparation

The photoanodes were prepared on glass plates (size 1.5 × 3 cm) cut out from a fluorine-doped tin oxide (FTO) glass sheet (thickness 2.2 mm, 13 Ω sq^−1^, Sigma-Aldrich, Poznań, Poland). Firstly, they were cleaned in a bath of detergent for cleaning of laboratory glass (TRILUX, Analab, Warsaw, Poland) diluted in distilled water. Next, they were soaked in pure distilled water and ethanol for 15 min each under ultrasonic conditions. Afterwards, a transparent and mesoporous titania layer was deposited by screen printing technique on dried plates (DN-HM02 screen-printer, Dyenamo, with a polyester screen of mesh count 250, Sefar, Stockholm, Sweden). The geometrical area of the TiO_2_ layer obtained by this method was 0.237 cm^2^. The paste used for the photoanode preparation is commercially available and contains particles in sizes of 28–31 nm (DN-GPS-30TS, Dyenamo, Stockholm, Sweden). The deposited TiO_2_ layer was gradually heated at 150 °C for 5 min, 300 °C for 5 min, and annealed at 450 °C for 60 min. The photoanodes were then dipped in 50 mM aqueous solution of TiCl_4_ for 30 min at 70 °C and rinsed with water. Finally, they were heated again at 150 °C for 5 min, 300 °C for 5 min, and 450 °C for 30 min. The thickness of the prepared mesoporous titania layer was about 2–3 μm [18]. The prepared photoanodes were immersed in one of the following solutions: (i) 0.3 mM ethanol solution of RuP dye (DN-S02, Dyenamo) or (ii) saturated ethanol solution of RuOEC catalyst (DN-S07, Dyenamo) at room temperature for about 16 h. In order to prepare the photoanode consisting of both RuP and RuOEC, sensitization of TiO_2_ was performed as described elsewhere [13] allowing 1 h for RuP and then 16 h for RuOEC.

### 2.2. Electrolyte Preparation

The following solutions were prepared by dilution in water (LC-MS Ultra CHROMASOLV^®^, Fluka, Bucharest, Romania): 0.07 M phosphate buffer (Fluka), 0.001 M hydrochloric acid (HCl, Sigma-Aldrich), 0.1 M potassium chloride (KCl, Mettler Toledo, Warsaw, Poland), and 0.00125 M EPA with different KCl concentrations (0.001, 0.01, and 0.1 M). The EPA solution was obtained by dissolving the acid in powder form in distilled water. All pH values of the solution were monitored using a pH meter (Mettler Toledo SevenCompact™, model S220) after the calibration procedure.

### 2.3. Stationary Absorption Characterization

A V-770 ultraviolet–visible–near-infrared (UV–Vis–NIR) spectrophotometer (Jasco, Inc., Mary’s Court Easton, MD, USA) equipped with a 150 mm integrating sphere (LN-925) was employed to record the stationary absorption spectra in the UV–Vis range. The samples were mounted in front of the integrating sphere to detect both transmitted and scattered light. The baselines for the unsensitized TiO_2_ electrodes were measured and subtracted from the absorption spectra of a dye-sensitized photoanode.

### 2.4. Photoelectrochemical Cell Setup

The photoelectrochemical cell consisted on three electrodes, with a photoanode acting as a working electrode. The counter electrode was a platinum wire, and the reference electrode was Ag/AgCl (1 M KCl). All measurements were conducted in the Dyenamo HT-holder (model DN-PH01).

The photocurrent generated by illuminating the photoanodes was recorded by a potentiostat/galvanostat (model M101 with a frequency response analyzer FRA32M module, Autolab, Cracow, Poland) coupled with a photoelectric spectrometer with a solar simulator (Photon Institute, Cracow, Poland). The time dependence of the photocurrent was recorded at a constant electrode polarization (0.236 or 0.736 V normal hydrogen electrode (NHE)). On the basis of the several measurements in the same configuration, we estimated the relative error of the photocurrent values to be 20% when the samples were from the same batch and measured on the same day, and up to 50% when the samples were from different batches and the measurements were made on different days.

The potentiostat/galvanostat was also employed for cyclic voltammetry (CV) and incident photon-to-current conversion efficiency (IPCE) spectra measurements, performed at an applied potential 0.236 V (vs. NHE) with monochromatic light only (without bias light). The sunlight conditions were maintained with the use of a Xe lamp with an AM 1.5 G spectral filter, while the irradiance was adjusted to 100 mW/cm^2^ using a calibrated cell (15151, ABET, Milford, CT, USA). In some experiments, a UV cut-off filter was introduced (λ ≥ 400 nm, THORLABS, FGL400S, Newton, NJ, USA).

## 3. Results and Discussion

### 3.1. Spectral Measurements

**Figure 1:**
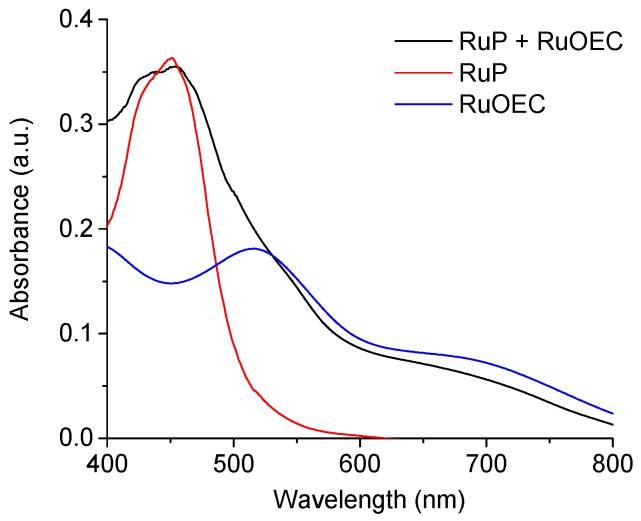
Absorption spectra of photoanodes sensitized in ruthenium(II)bis(2,2′-bipyridine)([2,2′-bipyridine]-4,4′-diylbis(phosphonic acid)) dibromide (RuP) for 1 h; ruthenium(II) tris(4-methylpyridine)(4-(4-(2,6-bis((l1-oxidanyl)carbonyl)pyridin-4-yl)phenyl) pyridine-2,6-dicarboxylic acid (RuOEC) for 16 h; and with RuP for 1 h and then RuOEC for 16 h. a.u.: Arbitrary units.

The representative absorption spectra of the photoanodes sensitized with RuP alone, RuOEC alone, and both dyes together (RuP + RuOEC) are shown in Figure 1. We determined the extinction coefficient for RuP in ethanol to be ≈14,250 M^−1^ cm^−1^ (455 nm), and that for RuOEC was ≈4300 M^−1^ cm^−1^ (530 nm), as previously reported [19]. The spectra recorded for dye layers on the TiO_2_ (Supplementary Figure S2), suggests that ca. 10% more molecules are adsorbed in the presence of RuP than of RuOEC, when TiO_2_ is sensitized with only one type of compound during the same time of sensitization. However, it was clearly observed that some of the absorbed RuP molecules were desorbed during the next step of photoanode sensitization in RuOEC solution (Figure 1, RuP + RuOEC). We assume that on the photoanode sensitized with both compounds there were approximately two times more adsorbed molecules of RuOEC than RuP, according to the intensity of relevant absorption bands (Figure 1).

**Figure 2:**
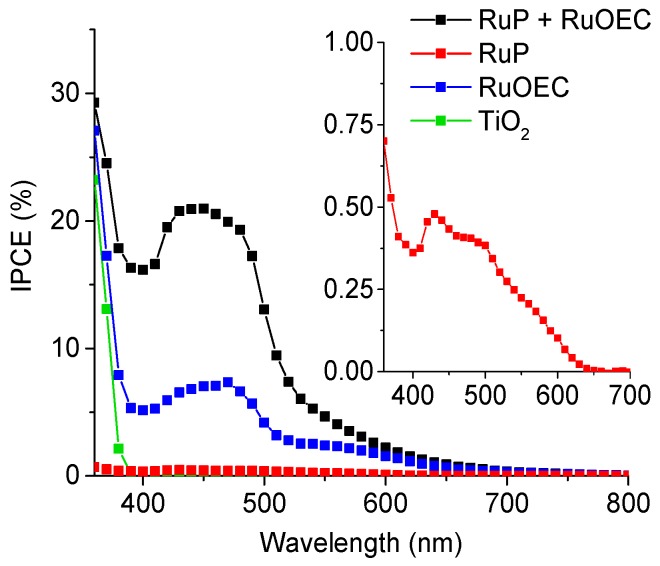
Incident photon-to-current efficiency (IPCE) spectra of selected water splitting systems with pure TiO_2_ and sensitized with RuP, RuOEC, or RuP + RuOEC. The electrolyte is HCl (pH ≈ 3). The inset shows an enlarged IPCE spectrum of TiO_2_ sensitized with RuP. The IPCE spectra were calculated based on the photocurrents measured within two seconds after switching the light on.

Incident photons to current efficiency spectra were recorded for complete photoelectrochemical cells in HCl (pH ≈ 3), for the photoanodes made with only pure TiO_2_ or TiO_2_ sensitized with RuP or RuOEC, and finally for those with both compounds (Figure 2). The collection of IPCE spectrum in a phosphate buffer (pH ≈ 7) was not feasible due to the very fast degradation of the system, since the measurement takes about 10 min. The spectrum of the photoanode with both the sensitizer and the catalyst reflects the absorption spectrum recorded for this photoanode (with TiO_2_ background subtracted, Figure 1). Besides the expected high photoresponse in the wavelength range corresponding to the maximum of absorption of RuP (≈450 nm), a high signal came from TiO_2_, especially below 400 nm, and also from RuOEC (≈530 nm). This indicates the charge separation processes related to the photoexcitation of each compound.

### 3.2. Chronoamperometry Studies

**Figure 3:**
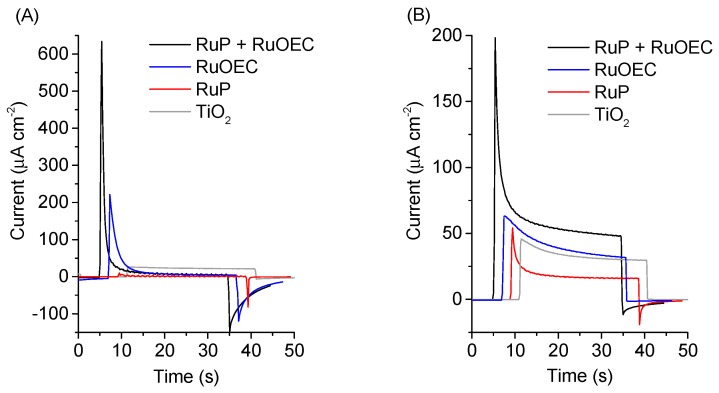
Photocurrent–time behavior of TiO_2_ photoanode pure and sensitized with RuP, RuOEC, or RuP + RuOEC in (**A**) HCl (pH ≈ 3) and (**B**) 0.07 M phosphate buffer solution (pH ≈ 7). The photoanodes were biased at 0.236 V vs. normal hydrogen electrode (NHE).

The parameters characterizing the transient photocurrent–time behavior observed for complete cells are collected in Table 1, and Supplementary Tables S1 and S2. The initial photocurrent *J*_0_ is the current response immediately after the light is switched on, for the first current trace cycle. The steady-state photocurrent *J*_stab_ was measured 30 s after the start of illumination. The dark photocurrent *J*_dark_ is the immediate photocurrent response after switching off the light. For clarity, multiple photocurrent traces are presented on the graphs with a 2 s time interval (Figure 3A,B).

The highest initial photocurrent response, *J*_0_, was recorded for a photoanode sensitized with both RuP + RuOEC (Supplementary Tables S1 and S2). The initial current peaks for the photoanodes with only pure (unsensitized) TiO_2_ are one order of magnitude lower than those for the photoanodes sensitized with RuP + RuOEC (Figure 3 and Supplementary Table S1). Moreover, for pure TiO_2_ in a phosphate buffer electrolyte, flat photocurrent traces were observed for the next light on–off cycles, therefore the initial current peak was only recorded in the first one (Supplementary Figure S3). The reason is that the structure of the titanium dioxide surface is permanently changed by the first illumination. However, in strongly acidic conditions most of the TiO_2_ surface is protonated, and therefore the photo-generated holes are accumulated near the surface and are unable to interact with water molecules upon illumination [20,21]. Therefore, a decrease in the photocurrent was observed even for the next on–off cycles. When the illumination was off, the system returned to equilibrium and low dark current was also observed (Supplementary Figure S3).

For the sensitized photoanodes the ratio *J*_stab_/*J*_0_ was pH- and Cl^−^ concentration-dependent, and it took the lowest values for the systems with low pH (Table 1). However, the highest *J*_stab_/*J*_0_ value of 0.21, obtained for the standard configuration with phosphate buffer, still corresponds to the unsatisfactory shape of the current decay (this decay is fast). Rapid decrease in the current amplitude was observed for the dye-sensitized photoanodes in all different electrolyte configurations, most probably due to the slow regeneration of the dyes (slow water oxidation process). The highest initial current peaks, *J*_0_, were recorded for the systems with HCl electrolyte (Table 1). However, in these conditions low steady current values were recorded (up to six times lower than in the configuration with a phosphate buffer solution (pH ≈ 7), Figure 3A,B), and noticeable dark currents reaching more than 100 µA/cm^2^ were also detected. As the catalytic water oxidizing reactions slow down when pH decreases, lower steady photocurrents are expected for HCl (pH ≈ 3) and EPA (pH ≈ 3) when compared to those for phosphate buffer (pH ≈ 7). It is worth noting that the photoanodes in the HCl electrolyte featured a ca. four times higher peak photocurrent response (*J*_0_) than EPA, although they had comparable pH (Table 1). On the other hand, the 0.001 M KCl addition to EPA (resulting in a Cl^−^ concentration equal to that of HCl) did not result in a noticeable increase in *J*_0_. A change in *J*_0_ was observed for higher KCl concentrations (e.g., *J*_0_ increased from 120 μA/cm^−2^ for 0.001 M KCl to 390 μA/cm^−2^ for 0.1 M KCl, Table 1).

It should be noted that from the point of view of water splitting efficiency, the photocurrents for different systems at the same bias voltage should be compared (e.g., at 0.236 V vs. NHE in Supplementary Table S1 or at 0.736 V vs. NHE in Supplementary Table S2). However, the height of photocurrent response may also be related to different pH, due to a shift of the oxidation/reduction peaks and a shift of the TiO_2_ conduction band. This problem will be discussed below when the photocurrent results at different biases are correlated with CV data.

### 3.3. Cyclic Voltammetry

**Figure 4:**
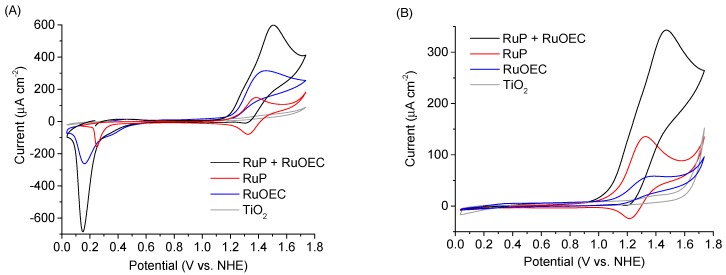
Cyclic voltammograms of TiO_2_ photoanode pure and sensitized with RuP, RuOEC, or RuP + RuOEC in (**A**) HCl (pH ≈ 3) and (**B**) 0.07 M phosphate buffer solution (pH ≈ 7), recorded in the dark. NHE: Normal hydrogen electrode.

**Figure 5:**
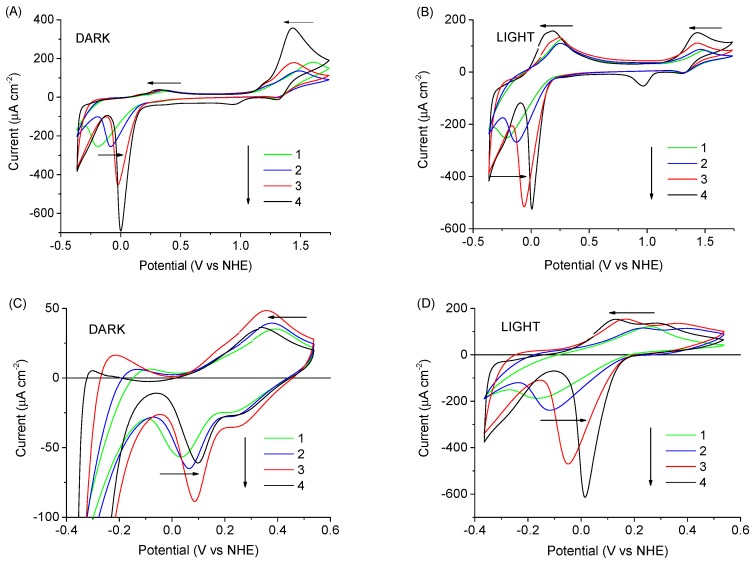
Cyclic voltammograms of TiO_2_ photoanode sensitized with RuP and RuOEC in EPA electrolyte containing KCl of different concentration: pure (1), 0.001 M KCl (2), 0.01 M KCl (3), and 0.1 M KCl (4). Scans in graphs (**A**,**C**) were recorded in dark condition, scans in (**B**,**D**) were recorded under 1 sun illumination with a UV cut-off filter (λ ≥ 400 nm).

The CV experiments were performed at the scan rate 50 mV/s. The open circuit potential was used as the start potential. A comparison of CV scans for photoanodes with different ruthenium compounds is presented in Figure 4 and Supplementary Figure S4, while Figure 5 shows the scans recorded for different Cl^−^ concentrations in electrolyte, and Supplementary Figure S5 presents the voltammograms of TiO_2_ photoanodes sensitized with RuP + RuOEC immersed in electrolytes of different pH (phosphate buffer, HCl, and KCl).

A steep oxidation peak current of catalytically oxidized water (above 1.0 V vs. NHE) was recorded for the photoanodes sensitized with RuP + RuOEC in a phosphate buffer (pH ≈ 7) and HCl (pH ≈ 3) (Figure 4A,B). This peak was not recorded for the configuration with unsensitized TiO_2_. The scans recorded for the RuP photoanode in HCl and phosphate buffer (Figure 4A,B) show the Ru(II)/Ru(III) oxidation/reduction peak [22]. However, the cathodic peak is not a perfect mirror reflection of the anodic one, as the latter is higher. It is worth mentioning that the potential required to oxidize ruthenium species is the insulating potential for TiO_2_. At such high potentials, the oxidation of the molecules most probably occurs through the electron/holes transfer from the FTO/TiO_2_ junction across the dye monolayer [23,24,25]. Due to the nature of electron transfer in such systems, the oxidation of molecules in cross-surface charge hopping is easier than reduction. Therefore, the reduction peak of RuP is less intensive than the oxidation peak. Moreover, the photoanode with both RuOEC catalyst and RuP dye exhibited intensive oxidation current response during the forward scan, while the corresponding cathodic peak was suppressed when compared to the RuP configuration. This indicates the gain of electrons which came from the water oxidation reaction, catalyzed by RuOEC.

During the reverse sweep, a pronounced cathodic peak was recorded below 0.3 V vs. NHE for the sensitized photoanodes in HCl (Figure 4A). This negative peak is assigned to the reduction of a dye and catalyst molecules, which are oxidized during the forward scan. It occurs when the potential is sufficiently negative to access the conduction band states of TiO_2_. In fact, the potential of the conduction band edge of TiO_2_ is about −0.2 V vs. NHE at pH 3, but most probably the trap states below the conduction band of TiO_2_ are responsible for the partial conduction at more positive potentials. The position of the peak changes for different electrolytes. For example, it was observed for the RuP + RuOEC photoanode in phosphate buffer (pH ≈ 7) and KCl (pH ≈ 5), but at more negative potentials (Supplementary Figure S5) than in HCl (pH ≈ 3), probably due to the negative shift of the TiO_2_ conduction band potential with increasing pH. However, it should be noted that the amplitude of this cathodic peak in the phosphate buffer was very small.

To check the effect of Cl^−^ on the electrochemical behavior, we compared the CV scans recorded in the potential range from −0.36 to 1.74 V vs. NHE for the RuP + RuOEC photoanodes in EPA (pH ≈ 3) electrolyte containing KCl in increasing concentration: 0.001, 0.01, and 0.1 M (Figure 5A). In cyclic voltammograms, regular changes in cathodic and anodic signals were observed in line with the varying KCl concentration. The position of the anodic oxidation peak clearly shifted to more negative values as the KCl concentration increased, while the position of the corresponding cathodic reduction remained almost the same. Moreover, the onset of oxidation did not shift (Figure 5A). This indicates the acceleration of the charge transfer related to the oxidation on the photoanode surface. In principle, this could also be due to a larger number of surface Ru species participating in charge transport on the surface, or the effective distance of the charge transport across the surface increasing with the increased Cl^−^ concentration. However, our preliminary time-resolved experiments indicated ≈40% faster electron injection and back electron transfer for the RuP samples in HCl than EPA electrolyte, which favors the first interpretation. Detailed time-resolved studies are planned in our future work. Additionally, the position of the cathodic peak at a low potential (below or close to 0 V vs. NHE) was shifted to more positive values. Cyclic voltammogram scans were also recorded for the same samples upon illumination with a UV cut-off filter (to ensure that observed effects were not influenced by TiO_2_ excitation, Figure 5B). Upon illumination, the intensity of the anodic peak below or close to 0 V (vs. NHE) increased, while the intensity of the anodic peak above 1.2 V (vs. NHE) decreased. This indicates that some parts of the dye molecules are oxidized at lower potentials before the potential reaches the redox potential of Ru(II)/Ru(III). Because these anodic peaks increase upon illumination, they might be related to the oxidation of the excited state.

The photoanodes in the same electrolytes configuration were studied by CV in a narrower potential range (−0.36 to 0.54 V vs. NHE) to avoid the intensive oxidation of ruthenium species induced by applying highly positive potential (Figure 5C,D). Only a small anodic peak assigned to the oxidation of excited dye molecules (up to 45 μA cm^−2^) and cathodic reduction peak (up to −80 μA cm^−2^ for RuP + RuOEC in EPA with 0.1 M KCl) were recorded in the dark (Figure 5C). This was probably due to small population of excited states, as the dark experiments were performed without strong 1 sun illumination, but at small ambient room light. The intensity of these peaks significantly increased when the photoanodes were illuminated with a sunlight simulator (Figure 5D). It is clearly visible that the peak-to-peak separation becomes smaller with increasing KCl concentration. On the basis of these observations, we conclude that the charge transfer on the photoanode surface increases with increasing KCl concentration, similarly as in the broader potential range in the dark described above. This phenomenon is not connected with the ionic strength changes, because for the HCl, for example, the most intensive anodic and cathodic peaks with small peak-to-peak separation were observed, although this electrolyte had the smallest ionic strength (≈0.001 M, Supplementary Table S3) from among all electrolytes. It is interesting to note that the oxidation of dyes was accelerated by the presence of Cl^−^ both in the dark when high potentials are applied (due to cross-surface electron hopping through dye monolayer to FTO), and under illumination in the limited potential range (due to electron injection to TiO_2_). This means that Cl^−^ have a positive influence on both charge transfer dynamics (electron hopping and injection).

The amplitudes in CV scans at certain potentials correlate with the distinctive shape of the current on–off trace, measured at the same applied bias voltage. The initial photocurrent peak *J*_0_ and the dark photocurrent *J*_dark_ recorded for RuP + RuOEC sample in HCl were the highest (*J*_0_ = 640 μA/cm^2^, *J*_dark_ = −140 μA/cm^2^), compared to those recorded in other the electrolytes (EPA, phosphate buffer, KCl; Table 1), all at 0.236 V vs. NHE. This is in line with the amplitude of the anodic peaks during forward scans upon illumination, which were the highest in HCl (Supplementary Figure S5B), as well as with the amplitude of the cathodic peaks in the dark during the reverse scan (S4A), both of which were around 0.2 V vs. NHE. Similarly, J_0_ and J_dark_ values were higher for RuP + RuOEC electrodes than for samples with only RuP or RuOEC (Supplementary Table S1), in agreement with amplitudes of the positive peaks under light (Supplementary Figure S5) and negative peaks in the dark (Figure 4A). However, it should be noted that the potential (0.236 V vs. NHE) at which chronoamperometry was recorded in this configuration is positioned within the range of observed anodic and cathodic peaks in CV scans recorded upon illumination (Figure S5B). When higher potential was applied the *J*_0_ and *J*_dark_ were substantially suppressed, as this potential is far from that of the CV peaks (Supplementary Table S2).

The EPA electrolyte with 0.1 M KCl featured the highest *J*_0_ among other mixtures with lower concentration of KCl, which also corresponds with specific observations in the CV measurements (e.g., smaller peak-to-peak separations). We conclude that the addition of Cl^−^ anions accelerates the charge transfer on a sensitized photoanode surface, and that it especially affects the hole transfer across the dye monolayer, resulting in a higher rate of RuP oxidation. The difference in photoelectrochemical behavior between photoanodes in 0.001 M HCl and EPA with 0.001 M KCl (e.g., the significant difference in the position of the cathodic current onset, Supplementary Table S3), although they were at the same Cl^−^ concentration and pH (≈3), may be explained by hindered contact of the photoanode with protons and Cl^−^, caused by the presence of EPA molecules and anions.

## 4. Conclusions

The presented studies are concerned with the electrochemical behavior of dye-sensitized photoanodes for water splitting in low pH electrolytes (pH ≈ 3) containing Cl^−^, in comparison to that in the standard phosphate buffer configuration (pH ≈ 7). The main focus was on the CV measurements performed for different potential ranges, with or without illumination of the photoanode. Analysis of the results leads to the conclusion that electron transfer on a dye-sensitized photoanode surface accelerates in the presence of Cl^−^. This effect is responsible for a significant increase in the initial anodic current recorded during chronoamperometric measurements. A correlation between photocurrent traces and CV measurements was observed, especially when the applied potential was near anodic and cathodic peaks in the CV scans recorded upon illumination, at which pronounced initial light and dark current peaks were recorded. The results observed for the same electrolyte (EPA at pH ≈ 3) with varying Cl^−^ concentrations clearly show that the acceleration of charge transfer depends on the Cl^−^ concentration. However, comparisons for different electrolytes (KCl at pH ≈ 5 and HCl at pH ≈ 3) indicate that the action of Cl^−^ also depends on the pH of the electrolyte and the presence of additional ions.

## Figures and Tables

**Table 1:** Parameters of the time traces of the photocurrent for the photoanode sensitized with both RuOEC and RuP.

Electrolyte	pH	*J*_0_ (μA/cm^2^)	*J*_stab_ (μA/cm^2^)	*J*_dark_ (μA/cm^2^)	*J*_stab_/*J*_0_
0.07 M Phosphate buffer	≈7	240	50	−20	0.21
0.1 M KCl	≈5	320	10	−20	0.03
0.001 M HCl	≈3	640	5	−140	0.01
EPA	≈3	140	7	−40	0.05
EPA + 0.001 M KCl		120	6	−40	0.05
EPA + 0.01 M KCl		200	7	−40	0.04
EPA + 0.1 M KCl		390	7	−40	0.02

*J*_0_: Initial photocurrent; *J*_stab_: Steady-state photocurrent; *J*_dark_: Dark photocurrent. The electrode area is taken as the geometric area.

## Supplementary Materials

The following are available online at http://www.mdpi.com/2313-7673/4/1/5/s1, Figure S1: Chemical structure of RuP and RuOEC, Figure S2: Absorption spectra of photoanodes sensitized in RuP (for 16 h) and RuOEC (for 16 h), Figure S3: Photocurrent–time behavior of TiO2 in HCl (pH ≈ 3) (1) and 0.07 M phosphate buffer solution (pH ≈ 7) (2), Figure S4: Cyclic voltammograms of TiO2 photoanode sensitized with RuP, RuOEC and RuP + RuOEC recorded under illumination in HCl electrolyte (pH ≈ 3), Figure S5: Cyclic voltammograms of TiO2 photoanode sensitized with RuP + RuOEC in electrolytes, Table S1: Parameters of the time traces of the photocurrent recorded at 0.236 V vs. NHE, Table S2: Parameters of the time traces of the photocurrent recorded at 0.736 V vs. NHE, Table S3: Onset of cathodic current peak obtained from CV scans recorded in dark conditions (from −0.36 to 1.74 V vs. NHE) for RuP + RuOEC photoanodes in different electrolytes.
