# Supplementary material for: The Effect of Chloride Anions on Charge Transfer in Dye-Sensitized Photoanodes for Water Splitting
Source: Biomimetics (Basel). 2019 Jan 16;4(1):5. doi: 10.3390/biomimetics4010005 (PMC6477621; doi:10.3390/biomimetics4010005)
Supplement: Supplementary file 1 [file biomimetics-04-00005-s001.pdf]

Biomimetics **2019**, *4*, 5

Supplementary Materials: The Effect of Chloride Anions on Charge Transfer in Dye-Sensitized Photoanodes for Water Splitting

Iwona Grądzka, Mateusz Gierszewski and Marcin Ziółek *

Quantum Electronics Laboratory, Faculty of Physics, Adam Mickiewicz University, ul. Umultowska 85, PL-61614 Poznań, Poland; iwona.gradzka@amu.edu.pl (I.G.); mgiersz@amu.edu.pl (M.G.)

* Correspondence: marziol@amu.edu.pl

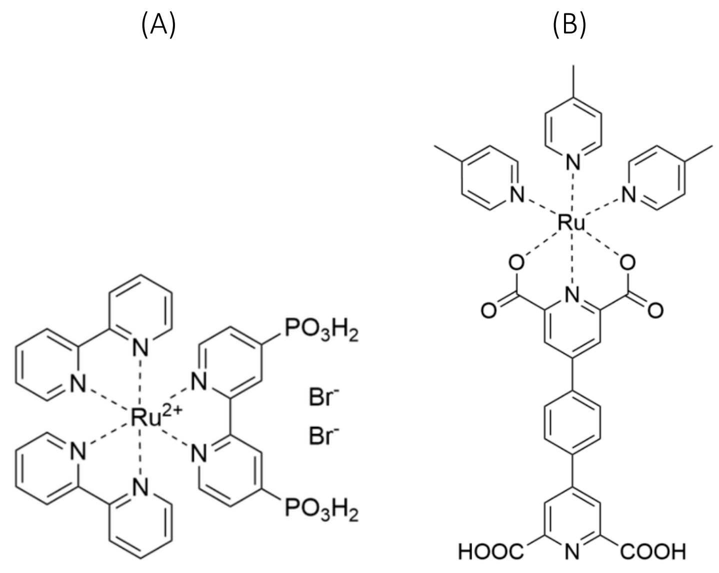

Figure S1. The chemical structures of (**A**) RuP and (**B**) RuOEC.

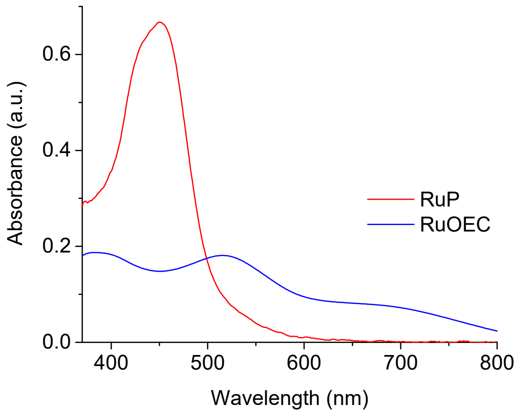

Figure S2. Absorption spectra of photoanodes sensitized in RuP (for 16h) and RuOEC (for 16h).

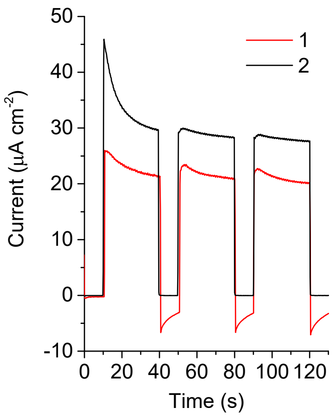

Figure S3. Photocurrent–time behaviour of TiO_2 in HCl (pH ≈3) (1) and 0.07 M phosphate buffer (pH ≈7) solution (2). The photoanodes were biased at 0.236 V vs. NHE.

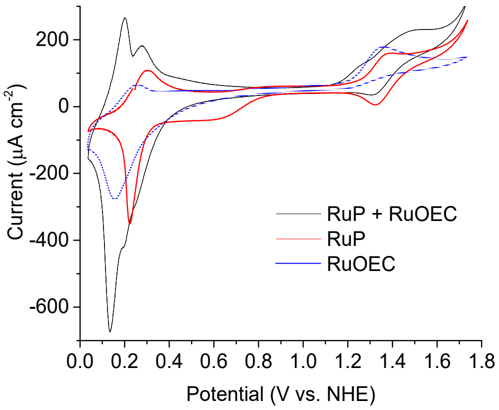

Figure S4. Cyclic voltammograms of TiO_2 photoanode sensitized with RuP, RuOEC and RuP + RuOEC recorded under illumination in HCl electrolyte (pH ≈3).

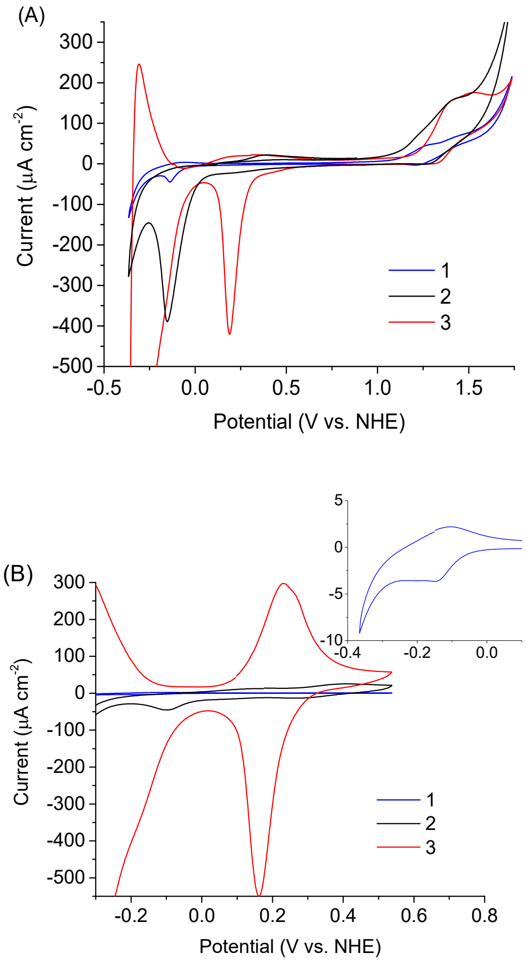

Figure S5. Cyclic voltammograms of TiO_2 photoanode sensitized with RuP + RuOEC in electrolytes: 0.07 M phosphate buffer (pH ≈7) (1), 0.1 M KCl (pH ≈5) (2), and 0.001 M HCl (pH ≈3) (3) recorded (**A**) in dark condition and (**B**) upon illumination when UV cut-off filter was used.

Table S1. Parameters of the time traces of the photocurrent recorded at 0.236 V vs. NHE.

Type of Photoanode	Electrolyte	pH	J_0 (μA/cm²)	J_{stab} (μA/cm²)	J_{dark} (μA/cm²)	J_{stab}/J_0
RuOEC	0.001 M HCl	≈3	220	5	−120	0.02
RuP			10	0	−80	0
RuP + RuOEC			640	5	−140	0.01
TiO_2			25	20	−5	0.80
RuOEC	0.07 M Phosphate buffer	≈7	65	30	0	0.46
RuP			55	15	−20	0.27
RuP + RuOEC			240	50	−20	0.20
TiO_2			45	30	0	0.67

J_0: Initial photocurrent; J_{stab}: Steady-state photocurrent; J_{dark}: Dark photocurrent; J_{stab}/J_0: Ratio for the photoanode sensitized with RuOEC, RuP and both.

Table S2. Parameters of the time traces of the photocurrent recorded at 0.736 V vs. NHE.

Type of Photoanode	Electrolyte	pH	J_0 (μA/cm^2)	J_{stab} (μA/cm^2)	J_{dark} (μA/cm^2)	J_{stab}/ J_0
RuOEC			290	40	−45	0.14
RuP	0.001 M HCl	≈3	70	10	−10	0.14
RuP + RuOEC			420	25	−5	0.06
RuOEC			25	25	0	1
RuP	0.07 M Phosphate buffer	≈7	85	30	−5	0.35
RuP + RuOEC			60	35	0	0.58

J_0: Initial photocurrent; J_{stab}: Steady-state photocurrent; J_{dark}: Dark photocurrent; J_{stab}/J_0: Ratio for the photoanode sensitized with RuOEC, RuP and both.

Table S3. Onset of cathodic current peak obtained from CV skans recorded in dark conditions (from −0.36 to 1.74 V vs. NHE) for RuP + RuOEC photoanodes in different electrolytes.

Electrolyte	Molar Ionic Strength (M)	pH	Onset of Cathodic Current (V vs. NHE) [1]
0.07 M Phosphate buffer	0.15	≈7	−0.06
0.1 M KCl	0.1	≈5	−0.02
0.001 M HCl	0.001	≈3	0.28
EPA	≈0.001	≈3	0.11
EPA + 0.001 M KCl	≈0.002		0.13
EPA + 0.01 M KCl	≈0.011		0.13
EPA + 0.1 M KCl	≈0.101		0.12

[1] Relative error ±0.01 V.
